# Potential Pathway of Nitrous Oxide Formation in Plants

**DOI:** 10.3389/fpls.2020.01177

**Published:** 2020-07-31

**Authors:** Arbindra Timilsina, Chuang Zhang, Bikram Pandey, Fiston Bizimana, Wenxu Dong, Chunsheng Hu

**Affiliations:** ^1^Key Laboratory of Agricultural Water Resources, Hebei Key Laboratory of Soil Ecology, Center for Agricultural Resources Research, Institute of Genetics and Developmental Biology, Chinese Academy of Sciences, Shijiazhuang, China; ^2^University of Chinese Academy of Sciences, Beijing, China; ^3^Key Laboratory of Mountain Ecological Restoration and Bio-resource Utilization and Ecological Restoration Biodiversity Conservation Key Laboratory of Sichuan Province, Chengdu Institute of Biology, Chinese Academy of Sciences, Chengdu, China

**Keywords:** anoxia, hypoxia, nitrate, nitrite, nitric oxide, nitrous oxide, mitochondrion

## Abstract

Plants can produce and emit nitrous oxide (N_2_O), a potent greenhouse gas, into the atmosphere, and several field-based studies have concluded that this gas is emitted at substantial amounts. However, the exact mechanisms of N_2_O production in plant cells are unknown. Several studies have hypothesised that plants might act as a medium to transport N_2_O produced by soil-inhabiting microorganisms. Contrarily, aseptically grown plants and axenic algal cells supplied with nitrate (NO_3_) are reported to emit N_2_O, indicating that it is produced inside plant cells by some unknown physiological phenomena. In this study, the possible sites, mechanisms, and enzymes involved in N_2_O production in plant cells are discussed. Based on the experimental evidence from various studies, we determined that N_2_O can be produced from nitric oxide (NO) in the mitochondria of plants. NO, a signaling molecule, is produced through oxidative and reductive pathways in eukaryotic cells. During hypoxia and anoxia, NO_3_ in the cytosol is metabolised to produce nitrite (NO_2_), which is reduced to form NO *via* the reductive pathway in the mitochondria. Under low oxygen condition, NO formed in the mitochondria is further reduced to N_2_O by the reduced form of cytochrome c oxidase (CcO). This pathway is active only when cells experience hypoxia or anoxia, and it may be involved in N_2_O formation in plants and soil-dwelling animals, as reported previously by several studies. NO can be toxic at a high concentration. Therefore, the reduction of NO to N_2_O in the mitochondria might protect the integrity of the mitochondria, and thus, protect the cell from the toxicity of NO accumulation under hypoxia and anoxia. As NO_3_ is a major source of nitrogen for plants and all plants may experience hypoxic and anoxic conditions owing to soil environmental factors, a significant global biogenic source of N_2_O may be its formation in plants *via* the proposed pathway.

## Introduction

Nitrous oxide (N_2_O) is a potent greenhouse gas, and its potential to increase global warming is approximately 300-fold higher than CO_2_ (Tian et al., 2018). Globally, the primary sources that release N_2_O into the atmosphere are soil, ocean, manure application, industries, and biomass burning (Thomson et al., 2012). The nitrification and denitrification processes, mainly mediated by certain groups of soil micro-organisms (Hu et al., 2015), account for more than two-thirds of its emission into the atmosphere (Thomson et al., 2012). These processes are considerably increased by human activities, leading to an increase in the production of N_2_O in the soil, and thus, its concentration in the atmosphere. Increased level of N_2_O in the atmosphere has significantly contributed to global warming (Tian et al., 2018); therefore, understanding the pathways of N_2_O formation in various sources is essential for mitigating these effects.

Key pathways involved in N_2_O production in microbes include nitrification, nitrifier denitrification, nitrification-coupled denitrification, and denitrification (Baggs, 2011; Hu et al., 2015; Tian et al., 2018). However, there seems to be a gap between source estimation and the global N_2_O budget, leading to a high level of uncertainty in the budget estimations (Davidson and Kanter, 2014). This gap may be because not all sources of N_2_O to the atmosphere are accounted for (Syakila and Kroeze, 2011). Therefore, it is necessary to understand all sources of N_2_O and underlying mechanisms to elucidate its global budget. The production of N_2_O in axenic microalgae (Weathers, 1984; Weathers and Niedzielski, 1986; Guieysse et al., 2013; Plouviez et al., 2017) and ascetically grown plants (Goshima et al., 1999; Hakata et al., 2003) indicates that it could be produced by higher organisms and that the processes might be different from those in micro-organisms. Algae and plants are not included as sources of N_2_O (Syakila and Kroeze, 2011; Lenhart et al., 2019; Plouviez et al., 2019), but they might be the missing sources of N_2_O, causing high uncertainties in the global budget.

The roles of plants in N_2_O emission to the atmosphere are diverse. Plants can not only modify soil characteristics and subsequently influence N_2_O production in the soil (Gao et al., 2019) but also produce it in significant amounts and release it to the atmosphere (Lenhart et al., 2019). Thus, understanding the pathway of N_2_O formation and the contribution of plants to total emission is essential to accurately estimate the global N_2_O budget. Several field-based studies have hypothesised that N_2_O emitted by plants is produced in soil by microorganisms (Chang et al., 1998; Rusch and Rennenberg, 1998; Machacova et al., 2013; Bowatte et al., 2014; Wen et al., 2017). In this theory, plants are considered just a medium to transport N_2_O produced by soil microorganisms; however, laboratory-based studies have provided clear evidence that plants produce and emit N_2_O although the underlying mechanisms are unknown (Goshima et al., 1999; Hakata et al., 2003; Lenhart et al., 2019). Therefore, N_2_O emitted by plants might originate from two sources, namely, the soil microorganisms and plants.

Studies, which have hypothesised that plant-emitted N_2_O is produced by soil microorganisms, have only measured the fluxes from plants and concluded that plant-emitted N_2_O might be produced by soil microorganisms. N_2_O produced in plant cells might also use the same pathway, that is, transpiration, to release it to the atmosphere. This raises the question whether measuring the fluxes alone provides substantial evidence to prove the hypothesis, because flux measurement methods can just estimate the emission of N_2_O and cannot distinguish the sources. More robust methods such as isotope studies would provide more insights to distinguish the sources of N_2_O. For examples, injecting ^15^N-N_2_O into the root zone and measuring the subsequent fluxes would elucidate whether plants are a medium for N_2_O transport or not. However, no study has injected ^15^N-labeled N_2_O into the soil zone and measured the subsequent N_2_O emission from plants. Moreover, more powerful tools such as site preference (SP) measurement would provide insights to distinguish the sources of N_2_O emitted by plants under field conditions.

In the natural environment, if plant emitted N2O constitute significant amount of both sources (soil micro-organisms and plant cells produced N2O), it will be highly challenging to distinguish the portion of the sources. A recent field experiment reported considerably lower N_2_O concentrations in soil water than in tree stems (Ward et al., 2019). Similarly, plants exposed to NH_4_ did not emit N_2_O despite the high rate of N_2_O production in the rhizosphere (Smart and Bloom, 2001), indicating that N_2_O emitted by plants might not be produced by soil microorganisms and that N_2_O emitted through transpiration might be a less significant process than N_2_O production in plants. Furthermore, the hypothesis that plants are just a conduit for soil microorganisms-produced N_2_O is not supported by a recent study of Lenhart et al. (2019). They provided new evidence that dual isotopocule fingerprints of N_2_O emitted by plants differed from that produced by all known microbial or chemical processes, indicating that plant-emitted N_2_O is produced in plant cells.

Although plants are known to produce N_2_O and emit it to the atmosphere, the exact mechanisms of N_2_O production in plant cells are unknown (Goshima et al., 1999; Hakata et al., 2003; Lenhart et al., 2019). This might be the reason that most studies on N_2_O fluxes in plants (Chang et al., 1998; Rusch and Rennenberg, 1998; Machacova et al., 2013; Bowatte et al., 2014; Wen et al., 2017) have hypothesised that plant parts act as a conduit for soil-produced N_2_O. Studies, which have claimed that plants could produce N_2_O, have not elucidated a possible production pathway. Therefore, the main objective of this study was to review the possible pathway of N_2_O formation in plant cells.

## Pathway of N_2_O Formation in Plant Cells

### Nitrate (NO_3_) as a Precursor for N_2_O Formation in Plant Cells

Nitrogen (N) is an essential macronutrient influencing cell metabolism (O’Brien et al., 2016). Plants can use several forms of N from the soil; however, NO_3_ and ammonium (NH_4_) are the major forms of inorganic N that are readily available for plant uptake (O’Brien et al., 2016; Hachiya and Sakakibara, 2017). NO_3_ is a major source of N for plants in agricultural and natural soils (von Wirén et al., 2000), due to its high soil concentration and diffusion coefficients, making it readily available to plant roots (Miller and Cramer, 2005). After absorption, NO_3_ is directly reduced in the root, transported to the leaf for reduction (Maathuis, 2009; Hachiya and Sakakibara, 2017), or stored in the vacuoles and remobilised when the external supply is limited (van der Leij, et al., 1998; Fan et al., 2007), making it an essential macronutrient in plant metabolism.

NO_3_ is a major source for N_2_O formation in both soil (Thomson et al., 2012) and plants (Goshima et al., 1999; Smart and Bloom, 2001; Hakata et al., 2003; Lenhart et al., 2015; Lenhart et al., 2019). Isotope labeling methods have demonstrated that plants as well as other eukaryotic organisms emit N_2_O, only when supplied with NO_3_. For example, when ^15^N-labeled NO_3_ was supplied as a source of N to various species of plants (Goshima et al., 1999; Smart and Bloom, 2001; Lenhart et al., 2019), lichens (Lenhart et al., 2015), and animals (Stief et al., 2009), ^15^N-labeled N_2_O was emitted, but when the N source was ^15^N-labeled NH_4_, there was no N_2_O emission. This evidence clearly shows that NO_3_ is the precursor of N_2_O in lichens, higher plants, and animals. Therefore, if plants were just a medium of transportation of soil-produced N_2_O as hypothesised by many studies (Chang et al., 1998; Rusch and Rennenberg, 1998; Machacova et al., 2013; Bowatte et al., 2014; Wen et al., 2017), aseptically grown plants would not have emitted N_2_O when supplied with NO_3_ (Goshima et al., 1999; Hakata et al., 2003). Similarly, if N_2_O emitted by plants is produced by microorganisms (nitrifying and denitrifying bacteria), NH_4_ supplementation should contribute to N_2_O emission from plants and aseptically grown plants should not emit N_2_O. As N_2_O was not emitted when plants were supplied with NH_4_ and aseptically grown plants emitted N_2_O (Goshima et al., 1999; Hakata et al., 2003), we predicted that NO_3_ metabolism in a cell might play a role in N_2_O formation in plants. Moreover, the processes in the soil microbial communities and higher organisms may be different, or the denitrification process may be common between microorganism and plants, as NO_3_ is the substrate for denitrification.

### Nitrite (NO_2_) Derived From NO_3_ Reduction Is the Precursor of N_2_O in Plant Cells

After the uptake of NO_3_ by plant roots, it is reduced to NO_2_ in plant cells by a cytosolic enzyme called nitrate reductase (NR) (Chamizo-Ampudia et al., 2017). However, in animals, NO_3_ from food is reduced by bacteria in the digestive tracks (Lundberg et al., 2008). Moreover, germ-free mice are reported to possess NR activity, and the activity is catalysed by xanthine oxidoreductase, which is significantly high in the gastrointestinal tissues, compared with other tissues (Jansson et al., 2008). Under normal conditions, NO_3_ absorbed by the roots is reduced to NO_2_ by the NR, and then nitrite reductase (NiR) catalyses the reduction of NO_2_ to NH_4_, which is incorporated into amino acids (Oliveira and Sodek, 2013; Plouviez et al., 2017). However, under hypoxic and anoxic conditions, root NO_3_ uptake increases with the activation level of NR (Botrel and Kaiser, 1997; Rockel et al., 2002; Morard et al., 2004; Horchani et al., 2010). Furthermore, NO_2_ accumulates in the cytoplasm of cells (Allègre et al., 2004; Morard et al., 2004), as both hypoxia and anoxia suppress the reduction of NO_2_ to NH_4_ (Botrel and Kaiser, 1997). A ^15^N isotope labeling study has showed that ^15^N-NO_2_ assimilation into amino acids is sharply reduced under hypoxic conditions (Oliveira and Sodek, 2013). The accumulated NO_2_ in the cytoplasm enters the mitochondria with the help of proteins in the chloroplast (Sugiura et al., 2007; Gupta and Igamberdiev, 2011). Moreover, mitochondrial inner membrane anion channels may import NO_2_ to the mitochondria (Gupta and Igamberdiev, 2011).

Not only NO_3_, but also NO_2_ is widely reported to be a precursor of N_2_O in eukaryotic organisms. Using ^15^N isotope labeling method, it has been demonstrated that NO_2_ is another precursor of N_2_O formation in plants and algal cells. For example, when ^15^N-labeled NO_2_ was supplied to aseptically grown tobacco plants (Goshima et al., 1999; Hakata et al., 2003) and algal systems (Weathers, 1984), they emitted ^15^N-N_2_O. Furthermore, axenic algae supplied with NO_2_ produced N_2_O (Guieysse et al., 2013; Plouviez et al., 2017). The enzyme NR has been proved to play a role in N_2_O production in plants. For example, when tobacco plants were supplied with NO_3_ and tungstate (NR inhibitor), N_2_O production was inhibited in the plants (Goshima et al., 1999). As NO_2_ also contributes to N_2_O production in plants (Goshima et al., 1999; Hakata et al., 2003), NR might indirectly be involved in N_2_O formation by catalysing the reduction of NO_3_ to NO_2_. A similar role of NR has been observed in algae (Plouviez et al., 2017). However, NiR-deficient transgenic plants and algae have been reported to produce N_2_O when supplied with NO_2_ (Hakata et al., 2003; Plouviez et al., 2017), and this suggests that the pathway of NO_2_ reduction to NH_4_ is not involved in N_2_O production in plants and algal cells. Additionally, NO_3_, NR, and NO_2_ are involved in N_2_O production in plants and algal cells, but NiR and NH_4_ are not involved in the N_2_O production pathway. This indicates that NO_2_ has to be transported to other cell organelles rather than plastid. Overall, the available evidence indicates that exogenous NO_2_ along with endogenous NO_2_ derived through NO_3_ reduction in the cytosol by NR plays a role in N_2_O formation in plant cells.

### Mitochondrial Reduction of Nitrite to NO

We previously discussed NO_3_ reduction to NO_2_ in the cytosol. NO has several essential roles in plant and animal cells (Wendehenne et al., 2001), and the conversion mechanisms of NO_2_ to NO are well established in eukaryotic cells. In plants, NO can be produced in the chloroplast, peroxisomes, and mitochondria by either oxidative or reductive pathways (Rőszer, 2012). The oxidative pathway is dependent on L-arginine, polyamine, or hydroxylamine, whereas the reductive pathway is dependent on NO_3_ and NO_2_ (Benamar et al., 2008; Lundberg et al., 2008; Gupta et al., 2011; Gupta and Igamberdiev, 2011; Astier et al., 2018). The oxidative pathway of NO formation is dominant when the oxygen supply to cells is sufficient, whereas the reductive path is dominant under hypoxic conditions. By shifting from the oxidative to reductive pathway, the cells maintain the level of NO along with the physiological and pathological oxygen and proton gradients (Lundberg et al., 2008). It may be essential to shift processes, as plants may experience hypoxia due to soil environmental conditions.

In plant cells, NO_2_ assimilation to NH_4_ by the NiR enzyme is a well-known pathway of NO_2_ metabolism. As NO_2_ addition can lead to N_2_O formation in plants (Goshima et al., 1999; Hakata et al., 2003) and NiR-deficient plants can produce N_2_O (Hakata et al., 2003), we suggest that NO_2_ is metabolised by another pathway in plants to produce N_2_O. Although the mechanisms of NO_2_ transport to the mitochondria are not precise, it is evident that the mitochondria are a site of reduction of NO_2_ to NO. For example, the mitochondria have been reported to reduce NO_2_ to NO under hypoxic and anoxic conditions in fungi (Kobayashi et al., 1996; Castello et al., 2006), algae (Tischner et al., 2004; Calatrava et al., 2017), plants (Gupta et al., 2005; Planchet et al., 2005; Benamar et al., 2008; Gupta and Kaiser, 2010), and animals (Ghafourifar and Richter, 1997; Giulivi et al., 1998; Kozlov et al., 1999; Castello et al., 2006; Ascenzi et al., 2014). However, the enzymes involved in the mitochondrial reduction of NO_2_ to NO are not clear. For example, mitochondria that lack NiR can reduce NO_2_ to NO in animals and plants (Gupta and Igamberdiev, 2011). Nitric oxide synthases (NOS) have been reported to be present in plant (Guo and Crawford, 2005) and animal mitochondria (Giulivi et al., 1998). However, the NOS activity in the mitochondria of plants is questioned (Moreau et al., 2008; Gupta and Kaiser, 2010). Tischner et al. (2004) identified an alternative oxidase (AOX) in the mitochondria as a catalyser of the reduction of NO_2_ to NO under anoxic conditions. The mitochondrial respiratory chain is responsible for NO production using NO_2_ as the substrate under low pH, hypoxic, or anoxic conditions (Castello et al., 2006). Mitochondrial and bacterial electron transport chains (ETCs) are involved in NO production from NO_2_ under hypoxic conditions than under normoxic conditions (Horchani et al., 2011). Under hypoxic conditions, NO_2_ is reduced to NO at complex III in the mitochondria of pea plants (Benamar et al., 2008). Ascenzi et al. (2014) reported cytochrome c in horse heart cells and bovine heart reduced NO_2_ to NO, and the activity was high under anoxic and acidic conditions (Basu et al., 2008). The mitochondrial molybdopterin enzymes in the reduced form catalyse the reduction of NO_2_ to NO, and the rate was increased when the pH was decreased from 7.5 to 6.5 (Jakobs et al., 2014; Sparacino-Watkins et al., 2014; Maia and Moura, 2015; Bender and Schwarz, 2018). Furthermore, cytochrome reductase in tobacco plants can reduce NO_2_ to NO (Alber et al., 2017). Although at the molecular level, the reductive pathway for NO formation is well documented, at the field scale, the emission of NO is less documented. For instance, when plants were supplied with NO_3_, NO was emitted under anoxic conditions (Klepper, 1987; Rockel et al., 2002). The leaf NO_2_ level and NO emission under anoxic conditions were significantly higher than those under normoxic conditions (Rockel et al., 2002). These findings suggest that NO_2_ can be reduced to NO in the mitochondria; however, the involvement of various enzymes within the mitochondria raises the question whether these enzymes catalyse the reduction process simultaneously or they function differently under varied cell environment.

### NO Conversion to N_2_O in the Mitochondria

NO is a signaling molecule in cells, and several studies have focused on its formation in the mitochondria. However, studies on the reduction of NO to N_2_O in the mitochondria are limited, although there is a strong indication that this process exists (Gupta and Igamberdiev, 2011). The inner membrane of the mitochondria has an enzyme called cytochrome c oxidase (CcO). The primary function of CcO is to reduce O_2_ to H_2_O (Collman et al., 2007; Blomberg and Ädelroth, 2018). Moreover, CcO has several other functions, such as the oxidisation of NO formed in the mitochondria to NO_2_ (Brudvig et al., 1980; Zhao et al., 1995; Pearce et al., 2002; Taylor and Moncada, 2010). Furthermore, the reduced form of CcO can catalyse the reduction of NO to N_2_O (Brudvig et al., 1980; Zhao et al., 1995). Thus, either oxidation or reduction of NO by CcO results in the metabolism of NO with safe end products. The similar properties of O_2_ and NO facilitate the binding of NO to CcO, and this activity is pronounced under oxygen-limited conditions (Ghafourifar and Cadenas, 2005). The mitochondrial electron transport chain (ETC) in axenic algae (*Chlamydomonas reinhardtii* and *Chlorella vulgaris*) catalyses the reduction of NO to N_2_O (Guieysse et al., 2013; Plouviez et al., 2017; Plouviez et al., 2019). CcO has some rudimentary nitric oxide reductase activity, and therefore, when NO is the substrate instead of O_2_, two molecules of NO yield N_2_O and H_2_O (Brudvig et al., 1980; Zhao et al., 1995; Koivisto et al., 1997; Igamberdiev et al., 2010; Blomberg and Ädelroth, 2018; Poderoso et al., 2019). It has also been proven isotopically that NO is reduced to N_2_O by CcO in higher organisms (Brudvig et al., 1980). As mitochondrial CcO has evolved from denitrifying enzymes, under hypoxic conditions in cells, the mitochondrial CcO can still reduce NO to N_2_O (Saraste, 1994; Saraste and Castresana, 1994). Furthermore, another enzyme in the mitochondria, that is, quinone of the ETC catalyses NO reduction to N_2_O (Alegria et al., 2004; Igamberdiev and Hill, 2009; Sanchez-Cruz and Alegría, 2009). Therefore, mitochondria can be a potent site of N_2_O formation under oxygen-limited conditions, and it should be a focus of future research.

**Figure 1:**
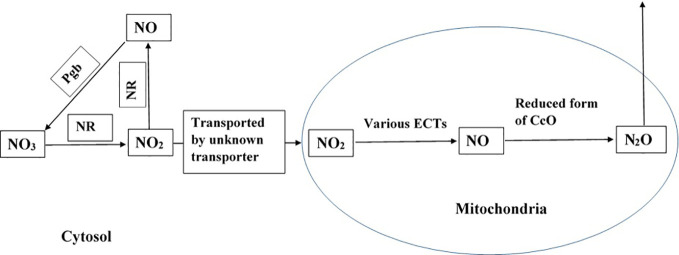
Potential pathway of N_2_O formation in plant cells. NR represents nitrate reductase and Pgb represents phytoglobin (Brudvig et al., 1980; Zhao et al., 1995; Goshima et al., 1999; Gupta and Kaiser, 2010; Guieysse et al., 2013; Plouviez et al., 2017). The pathway is active in presence of NO_3_ and NO_2_, and when plants experience hypoxia and anoxia.

Similar to the observations in plants, macrofauna and earthworms are also found to emit N_2_O when supplied with NO_3_ and under O_2_-limited conditions (Horn et al., 2003; Stief et al., 2009). Earthworms do not produce N_2_O when supplied NH_4_ (Horn et al., 2003). Moreover, in other studies, listed in **Table 1**, when ^15^N-labeled NH_4_ was used as a substrate, there was no N_2_O emission. This shows that NO_3_ metabolism at the cellular level produces N_2_O in both plants and animals. As described above, the ETC in (**Figure 1**) mitochondria can reduce NO to N_2_O under less oxic conditions, which suggests that N_2_O emitted by earthworms and macrofauna (Horn et al., 2003; Stief et al., 2009) might also be produced from hypoxic mitochondria. The gut of insects has a hypoxic environment (Johnson and Barbehenn, 2000), which may explain the higher level of N_2_O production in the gut (Stief et al., 2009). Moreover, axenic algae supplied with NO_2_ produced significantly higher levels of N_2_O under dark conditions than under light conditions (Guieysse et al., 2013; Plouviez et al., 2017). The low emission of N_2_O under light conditions may be due to the supply of photosynthetic O_2_ to the cells.

**Table 1:** Compilation of the substrates, mediums and products that used labeled N sources and their subsequent measurements of N_2_O emissions.

	Substrate	Medium	Product	Reference
1.	^15^N labeled NO_3_ ^15^N labeled NH_4_	Aseptically grown tobacco plants	^15^N labeled N_2_O No N_2_O emission	Goshima et al. (1999)
2.	^15^N labeled NO_3_ ^15^N labeled NH_4_	Lichen	^15^N labeled N_2_O No N_2_O emission	Lenhart et al. (2015)
3.	^15^N labeled NO_3_ ^15^N labeled NH_4_	Wheat plant	^15^N labeled N_2_O No N_2_O emission	Smart and Bloom (2001)
4.	^15^N labeled NO_3_	Soybean plant	^15^N labeled NO and N_2_O	Dean and Harper (1986)
5.	^15^N labeled NO_3_	Macro fauna	^15^N labeled N_2_O	Stief et al. (2009)
6.	^15^N labeled NO_2_	Tobacco plant	^15^N labeled N_2_O	Goshima et al. (1999)
7.	^15^N labeled NO_2_	Aseptically grown tobacco plant	^15^N labeled N_2_O	Hakata et al. (2003)
8.	^15^N labeled NO_2_	Axenic algae	^15^N labeled N_2_O	Weathers (1984)
9.	^15^N labeled NO ^14^N labeled NO	Reduced form of beef heart cytochrome c oxidase (CcO)	^15^N labeled N_2_O ^14^N labeled N_2_O	Brudvig et al. (1980)

Based on experimental evidence gathered from various studies, we propose that the reductive pathway of NO formation in the mitochondria and further reduction of NO by the mitochondrial ETCs contributes to the formation of N_2_O (in eukaryotic cells, as presented in **Figure 1**). The process is catalysed by various enzymes, and it might be pronounced under hypoxic and anoxic conditions but not under normoxic conditions. The proposed pathway is further supported by the existence of a denitrifying pathway, and the associated enzymes and genes in *Globobulimina* species and the localisation of enzymes in the mitochondria (Woehle et al., 2018). As higher animals possess well developed respiratory and circulatory systems that transport O_2_, they may not experience hypoxia. However, plants lack such sophisticated systems to transport O_2_ (Voesenek et al., 2016), and therefore, may experience hypoxia and anoxia that favour N_2_O formation. Field studies have reported high N_2_O emission from plants under flooded conditions (Rusch and Rennenberg, 1998; Machacova et al., 2013), suggesting the role of hypoxia and anoxia in N_2_O formation in plants.

## Significance of N_2_O Formation *via* the NO_3_-NO_2_-NO Pathway in Plants

NO_3_ is not only a major nutrient in plant cells but also a signaling molecule (Zhao et al., 2018). Several studies have reported that NO_3_ plays a role in hypoxia tolerance. For example, NO_3_ maintains the growth of plants under oxygen-limited conditions, and its absence disturbs plant growth (Horchani et al., 2010). Anoxia tolerance of tomato plant is enhanced by nitrate reduction (Allègre et al., 2004). Moreover, anoxia strongly induces NR activity and the induced NR activity prevents pH from dropping to life-threatening levels (Allègre et al., 2004). NO_3_ nutrition in plants decreases the total respiration rate and reactive oxygen species levels (Wany et al., 2019), but increases ATP production under hypoxic conditions (Stoimenova et al., 2007; Wany et al., 2019). Under oxygen-limited conditions, NO_3_ protects the ultrastructure of mitochondria (Vartapetian et al., 2003). The addition of NO_3_ to the root zone of plants released significantly less amount of ethanol compared with roots supplied with NH_4_ under hypoxic conditions (Oliveira et al., 2013). This suggests NO_3_ plays an important role to decrease alcoholic fermentative metabolism in plants during hypoxia (Oliveira et al., 2013). Overall, these findings suggest that NO_3_ and NR play an important role to maintain the integrity of plant cells under oxygen-limited conditions.

NO_2_ is also reported to play important roles under oxygen-limited conditions in plants. Benamar et al. (2008) found that NO_2_-dependent NO production in the mitochondria can regulate surrounding O_2_ level. Moreover, plant mitochondria can synthesise ATP under anaerobic conditions when supplied with NO_2_ (Stoimenova et al., 2007; Gupta et al., 2016). The supply of NO_2_ decreased lipid peroxidation and reactive oxygen species formation (Gupta et al., 2016). The absence of NO_2_ as a terminal acceptor for ETC during hypoxia leads to mitochondrial depolarisation (Gupta et al., 2016). NO_2_ supplemented roots released significantly less amount of fermentative ethanol during hypoxia than NH_4_-supplemented roots (Oliveira et al., 2012; Oliveira et al., 2013), suggesting the vital role of NO_2_ in plants to survive under oxygen-limited conditions.

**Figure 2:**
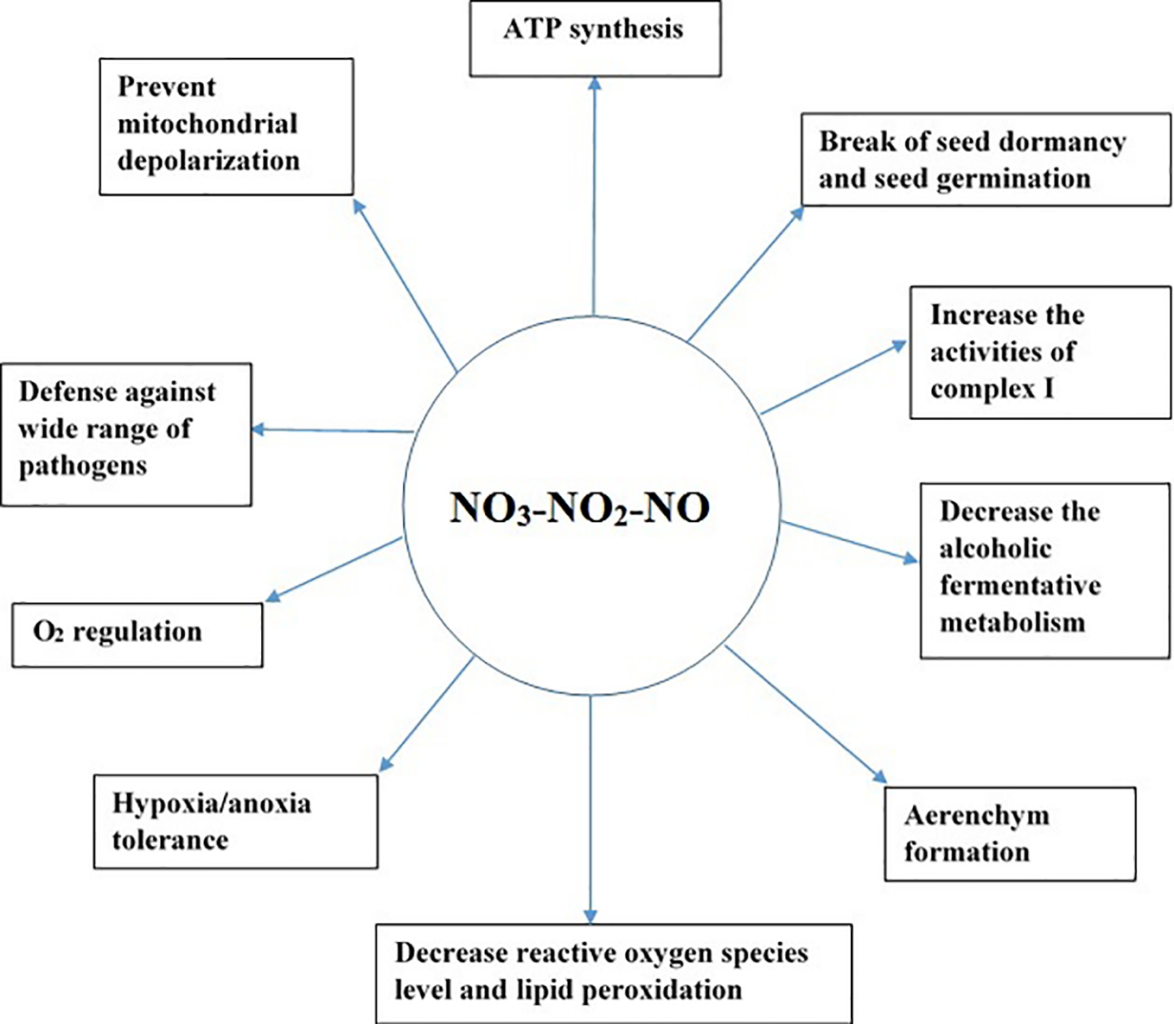
Significance of the NO_3_-NO_2_-NO pathway in plants under hypoxic and anoxic conditions.

NO helps plants to cope under several environmental stresses. For example, NO is essential for the homeostasis of O_2_ level in plants under oxygen-limited conditions (Gupta and Igamberdiev, 2011). NO production in the mitochondria has several implications in plants as illustrated in **Figure 2**. For example, NO can break seed dormancy and stimulate seed germination in plants (Beligni and Lamattina, 2000; Bethke et al., 2004). Similarly, under hypoxic stress, NO is vital for the formation of aerenchyma in the roots (Wany et al., 2017). NO production in the mitochondria under low-oxygen conditions can help in ATP synthesis, preventing excessive depletion of energy (Stoimenova et al., 2007). NO_3_-NO_2_-dependent NO production in plant roots decreases fermentative ethanol production during hypoxia (Oliveira et al., 2012; Oliveira et al., 2013).

Although NO has been well established as a signaling molecule, its high concentration in cells leads to cell death (Boscá and Hortelano, 1999; Brown and Borutaite, 2002). Therefore, it is critical to regulate its concentration in cells, as a higher amount of NO is formed under hypoxic and anoxic conditions. Two mechanisms are reported to occur in the mitochondria to detoxify the high amount of NO formed, namely, oxidation of NO to NO_2_ during normoxia (Cooper, 2002; Taylor and Moncada, 2010) and reduction of NO to N_2_O during hypoxia (Cooper, 2002). As both products of NO metabolism in the mitochondria, that is, NO_2_ and N_2_O, are non-toxic, their formation might play a protective role in the mitochondria. If hypoxia-induced NO production in cells is high, it can cause DNA fragmentation, leading to cell death; however, if NO is scavenged, it can reduce DNA fragmentation (Wany et al., 2017). Therefore, scavenging of NO is essential to protect cells from high NO toxicity. Phytoglobins are reported to scavenge NO in the cytosol (Igamberdiev et al., 2010). Additionally, purified mitochondria have been reported to scavenge exogenous NO (Gupta et al., 2005; de Oliveira et al., 2008; Wulff et al., 2009; Gupta and Kaiser, 2010). Furthermore, the addition of NADH as an electron donor increased NO scavenging by the mitochondria (Gupta et al., 2005; de Oliveira et al., 2008; Wulff et al., 2009; Gupta et al., 2016), indicating that the mitochondria have a protective mechanism to detoxify the excess NO formed. NADH might act as an electron donor to reduce cytochrome c oxidase, leading to an increase in NO scavenging in purified mitochondria. As discussed in our proposed pathway of N_2_O formation in the mitochondria, the conversion of NO to N_2_O by the reduced form of CcO might be the potential pathway regulating excessive NO formed under oxygen-limited conditions in the mitochondria. Mitochondria are not only a source of NO, but also an important sink and target of NO (Igamberdiev et al., 2014), and long-term exposure of mitochondria to NO can lead to the dysfunction of mitochondria (Brown and Borutaite, 2002). Although the NO_3_-NO_2_-NO pathway has several roles (**Figure 2**) in plants during hypoxia and anoxia, NO accumulation at higher level is toxic to cells (Brown and Borutaite, 2002). Therefore, N_2_O formation in the mitochondria *via* the NO_3_-NO_2_-NO pathway might be a strategy to protect cells and mitochondrial components from excessive NO formed under oxygen-limited conditions. Therefore, at molecular level, further research should focus on measuring NO and N_2_O from isolated mitochondria to obtain more insights on mitochondria’s role in scavenging excess NO during hypoxia and anoxia.

## Do Plant Cells Reduce N_2_O to N_2_?

The last two enzymes of the denitrification process, namely, nitric oxide reductase (NOR) and nitrous oxide reductase (N_2_OR), merged to form CcO (Saraste and Castresana, 1994; Stanton et al., 2018). Moreover, the copper site in bacterial N_2_OR is similar to the CuA site in CcO (Kroneck, 2018). Many catalytic properties of CcO from denitrifying bacteria (*Paracoccus denitrificans*) and eukaryotic organisms are similar (Ludwig, 1987; Kadenbach et al., 1991). As eukaryotic mitochondrion is considered to be evolved from *P. denitrificans*, a denitrifying bacterium (John and Whatley, 1975), it may still possess rudimentary denitrification properties. Although during the evolution most of genes of the bacterium transferred to the nucleus, few remained in the mitochondrial DNA including genes of CcO (Kadenbach et al., 1991). Therefore, it may be possible that CcO of higher organisms might also possess similar properties like that of its ancestor, *P. denitrificans*. The significant negative relationship between N_2_O consumption and CO_2_ respiration rates in plants and lichens (Machacova et al., 2017) suggests that mitochondria are the possible site of N_2_O consumption. This N_2_O consumption observed in these eukaryotes might be at the site of CcO, as this enzyme is formed from the last two enzymes of denitrification. There are also reports of emission of ^15^N-labeled N_2_ from wheat crops supplied with ^15^N-labeled NO_2_ (Vanecko and Varner, 1955), suggesting that under certain cell conditions, mitochondria may also metabolise N_2_O to N_2_. However, to date, N_2_ emission from plants is less reported. It may be due to the advanced systems of O_2_ regulation in plants, and this might inhibit the complete process of denitrification. A recent study, which measured N_2_O and N_2_ emission from soil–plant systems, showed that N_2_O and N_2_ emitted by NO_3_-rich soil–plant systems was three times higher than that by NH_4_-supplemented soil–plant systems and bare soil (Senbayram et al., 2020), and this suggests that the possible role of N_2_O and N_2_ production in plants. Further experiments at the molecular level (mitochondria) are needed to explore the reason for the significant negative relation between N_2_O emission and respiration rate in plants and lichen, as reported by Machacova et al. (2017).

## Conclusions

To cope with the problems of global warming and ozone layer depletion, a good understanding of N_2_O formation processes in various source is critical. Therefore, the N_2_O formation process in plants is a matter of concern. The reductive pathway of NO formation in the mitochondria along with further reduction of NO by ETC is a possible pathway of N_2_O formation in plants. Considering available evidence, we conclude that there is strong possibility that plant cells produce N_2_O in the mitochondria under hypoxic and anoxic conditions. The theory that plants are only a conduit for N_2_O produced by soil-inhabiting microorganisms might be an ambiguous explanation. The root zone may sense hypoxia and anoxia due to the soil environmental conditions, which may favour N_2_O formation in the root mitochondria. As some studies have shown that N_2_O emission from tree stems is higher than that from soils in natural habitats (Welch et al., 2019), the proposed pathway of N_2_O formation in plants might play a significant role in understanding N cycling in eukaryotic organisms and the global N_2_O budget. Furthermore, we have highlighted the reduction of NO to N_2_O in the mitochondria, and therefore, it would be valuable to reassess the role of mitochondrial ETC under both hypoxic and anoxic conditions. Although, N_2_O is a potent greenhouse gas ,its formation in the mitochondria might help to protect the integrity of the mitochondria and protect cells from the toxicity of NO accumulation during hypoxia.

## Author Contributions

AT wrote the manuscript. CH supervised the whole work. CZ, BP, FB, and WD commented on the manuscript. All authors contributed to the article and approved the submitted version.

## Funding

This work was funded by the National Natural Science Foundation of China (no. 41530859) and the National Key Research and Development Program of China (2016YFD0800102-4 and 2018YFC0213300-01).

## Conflict of Interest

The authors declare that the research was conducted in the absence of any commercial or financial relationships that could be construed as a potential conflict of interest.

## References

[B1] Alber N. A. Sivanesan H. Vanlerberghe G. C. (2017). The occurrence and control of nitric oxide generation by the plant mitochondrial electron transport chain. Plant Cell Environ. 40, 1074–1085. 10.1111/pce.12884 27987212

[B2] Alegria A. E. Sanchez S. Quintana I. (2004). Quinone-enhanced ascorbate reduction of nitric oxide: role of quinone redox potential. Free Radical Res. 38, 1107–1112. 10.1080/10715760400009852 15512799

[B3] Allègre A. Silvestre J. Morard P. Kallerhoff J. Pinelli E. (2004). Nitrate reductase regulation in tomato roots by exogenous nitrate: a possible role in tolerance to long-term root anoxia. J. Exp. Bot. 55, 2625–2634. 10.1093/jxb/erh258 15475378

[B4] Ascenzi P. Marino M. Polticelli F. Santucci R. Coletta M. (2014). Cardiolipin modulates allosterically the nitrite reductase activity of horse heart cytochrome c. J. Biol. Inorg. Chem. 19, 1195–1201. 10.1007/s00775-014-1175-9 24969400

[B5] Astier J. Gross I. Durner J. (2018). Nitric oxide production in plants: an update. J. Exp. Bot. 69, 3401–3411. 10.1093/jxb/erx420 29240949

[B6] Baggs E. M. (2011). Soil microbial sources of nitrous oxide: recent advances in knowledge, emerging challenges and future direction. Curr. Opin. Env. Sust. 3, 321–327. 10.1016/j.cosust.2011.08.011

[B7] Basu S. Azarova N. A. Font M. D. King S. B. Hogg N. Gladwin M. T. (2008). Nitrite reductase activity of cytochrome c. J. Biol. Inorg. Chem. 283, 32590–32597. 10.1074/jbc.M806934200 PMC2583304 18820338

[B8] Beligni M. V. Lamattina L. (2000). Nitric oxide stimulates seed germination and de-etiolation, and inhibits hypocotyl elongation, three light-inducible responses in plants. Planta 210 (2), 215–221. 10.1007/PL00008128 10664127

[B9] Benamar A. Rolletschek H. Borisjuk L. Avelange-Macherel M. H. Curien G. Mostefai H. A. (2008). Nitrite–nitric oxide control of mitochondrial respiration at the frontier of anoxia. BBA-Bioenergetics 1777, 1268–1275. 10.1016/j.bbabio.2008.06.002 18602886

[B10] Bender D. Schwarz G. (2018). Nitrite-dependent nitric oxide synthesis by molybdenum enzymes. FEBS Lett. 592, 2126–2139. 10.1002/1873-3468.13089 29749013

[B11] Bethke P. C. Gubler F. Jacobsen J. V. Jones R. L. (2004). Dormancy of Arabidopsis seeds and barley grains can be broken by nitric oxide. Planta 219, 847–855. 10.1007/s00425-004-1282-x 15133666

[B12] Blomberg M. R. Ädelroth P. (2018). Mechanisms for enzymatic reduction of nitric oxide to nitrous oxide-A comparison between nitric oxide reductase and cytochrome c oxidase. BBA-Bioenergetics 1859, 1223–1234. 10.1016/j.bbabio.2018.09.368 30248312

[B13] Boscá L. Hortelano S. (1999). Mechanisms of nitric oxide-dependent apoptosis: involvement of mitochondrial mediators. Cell Signal. 11, 239–244. 10.1016/S0898-6568(98)00064-3 10372801

[B14] Botrel A. Kaiser W. M. (1997). Nitrate reductase activation state in barley roots in relation to the energy and carbohydrate status. Planta 201, 496–501. 10.1007/s004250050094 9151451

[B15] Bowatte S. Newton P. C. Theobald P. Brock S. Hunt C. Lieffering M. (2014). Emissions of nitrous oxide from the leaves of grasses. Plant Soil 374, 275–283. 10.1007/s11104-013-1879-6

[B16] Brown G. C. Borutaite V. (2002). Nitric oxide inhibition of mitochondrial respiration and its role in cell death. Free Radical Bio. Med. 33, 1440–1450. 10.1016/S0891-5849(02)01112-7 12446201

[B17] Brudvig G. W. Stevens T. H. Chan S. II (1980). Reactions of nitric oxide with cytochrome c oxidase. Biochemistry 19, 5275–5285. 10.1021/bi00564a020 6255988

[B18] Calatrava V. Chamizo-Ampudia A. Sanz-Luque E. Ocaña-Calahorro F. Llamas A. Fernandez E. (2017). How Chlamydomonas handles nitrate and the nitric oxide cycle. J. Exp. Bot. 68, 2593–2602. 10.1093/jxb/erw507 28201747

[B19] Castello P. R. David P. S. McClure T. Crook Z. Poyton R. O. (2006). Mitochondrial cytochrome oxidase produces nitric oxide under hypoxic conditions: implications for oxygen sensing and hypoxic signaling in eukaryotes. Cell Metab. 3, 277–287. 10.1016/j.cmet.2006.02.011 16581005

[B20] Chamizo-Ampudia A. Sanz-Luque E. Llamas A. Galvan A. Fernandez E. (2017). Nitrate reductase regulates plant nitric oxide homeostasis. Trends Plant Sci. 22, 163–174. 10.1016/j.tplants.2016.12.001 28065651

[B21] Chang C. Janzen H. H. Nakonechny E. M. Cho C. M. (1998). Nitrous oxide emission through plants. Soil Sci. Soc Am. J. 62, 35–38. 10.2136/sssaj1998.03615995006200010005x

[B22] Collman J. P. Devaraj N. K. Decréau R. A. Yang Y. Yan Y. L. Ebina W. (2007). A cytochrome c oxidase model catalyzes oxygen to water reduction under rate-limiting electron flux. Science 315, 1565–1568. 10.1126/science.1135844 17363671 PMC3064436

[B23] Cooper C. E. (2002). Nitric oxide and cytochrome oxidase: substrate, inhibitor or effector? Trends Biochem. Sci. 27, 33–39. 10.1016/S0968-0004(01)02035-7 11796222

[B24] Davidson E. A. Kanter D. (2014). Inventories and scenarios of nitrous oxide emissions. Environ. Res. Lett. 9:105012. 10.1088/1748-9326/9/10/105012

[B25] de Oliveira H. C. Wulff A. Saviani E. E. Salgado I. (2008). Nitric oxide degradation by potato tuber mitochondria: evidence for the involvement of external NAD(P)H dehydrogenases. BBA-Bioenergetics 1777, 470–476. 10.1016/j.bbabio.2008.02.006 18371295

[B26] Dean J. V. Harper J. E. (1986). Nitric oxide and nitrous oxide production by soybean and winged bean during the in vivo nitrate reductase assay. Plant Physiol. 82, 718–723. 10.1104/pp.82.3.718 16665099 PMC1056196

[B27] Fan X. Jia L. Li Y. Smith S. J. Miller A. J. Shen Q. (2007). Comparing nitrate storage and remobilization in two rice cultivars that differ in their nitrogen use efficiency. J. Exp. Bot. 58 (7), 1729–1740. 10.1093/jxb/erm033 17351248

[B28] Gao G. F. Li P. F. Zhong J. X. Shen Z. J. Chen J. Li Y. T. (2019). *Spartina alterniflora* invasion alters soil bacterial communities and enhances soil N_2_O emissions by stimulating soil denitrification in mangrove wetland. Sci. Total Environ. 653, 231–240. 10.1016/j.scitotenv.2018.10.277 30412868

[B29] Ghafourifar P. Cadenas E. (2005). Mitochondrial nitric oxide synthase. Trends Pharmacol. Sci. 26, 190–195. 10.1016/j.tips.2005.02.005 15808343

[B30] Ghafourifar P. Richter C. (1997). Nitric oxide synthase activity in mitochondria. FEBS Lett. 418, 291–296. 10.1016/S0014-5793(97)01397-5 9428730

[B31] Giulivi C. Poderoso J. J. Boveris A. (1998). Production of nitric oxide by mitochondria. J. Biol. Chem. 273, 11038–11043. 10.1074/jbc.273.18.11038 9556586

[B32] Goshima N. Mukai T. Suemori M. Takahashi M. Caboche M. Morikawa H. (1999). Emission of nitrous oxide (N_2_O) from transgenic tobacco expressing antisense NiR mRNA. Plant J. 19, 75–80. 10.1046/j.1365-313X.1999.00494.x 10417728

[B33] Guieysse B. Plouviez M. Coilhac M. Cazali L. (2013). Nitrous Oxide (N_2_O) production in axenic Chlorella vulgaris microalgae cultures: evidence, putative pathways, and potential environmental impacts. Biogeosciences 10, 6737–6746. 10.5194/bg-10-6737-2013

[B34] Guo F. Q. Crawford N. M. (2005). Arabidopsis nitric oxide synthase1 is targeted to mitochondria and protects against oxidative damage and dark-induced senescence. Plant Cell 17, 3436–3450. 10.1105/tpc.105.037770 16272429 PMC1315380

[B35] Gupta K. J. Igamberdiev A. U. (2011). The anoxic plant mitochondrion as a nitrite: NO reductase. Mitochondrion 11, 537–543. 10.1016/j.mito.2011.03.005 21406251

[B36] Gupta K. J. Kaiser W. M. (2010). Production and scavenging of nitric oxide by barley root mitochondria. Plant Cell Physiol. 51, 576–584. 10.1093/pcp/pcq022 20185408

[B37] Gupta K. J. Stoimenova M. Kaiser W. M. (2005). In higher plants, only root mitochondria, but not leaf mitochondria reduce nitrite to NO, in vitro and in situ. J. Exp. Bot. 56, 2601–2609. 10.1093/jxb/eri252 16131511

[B38] Gupta K. J. Fernie A. R. Kaiser W. M. van Dongen J. T. (2011). On the origins of nitric oxide. Trends Plant Sci. 16, 160–168. 10.1016/j.tplants.2010.11.007 21185769

[B39] Gupta A. K. Kumari A. Mishra S. Wany A. Gupta K. J. (2016). “The functional role of nitric oxide in plant mitochondrial metabolism,” in Advances in Botanical Research, vol. 77 (Cambridge, MA: Academic Press), 145–163. 10.1016/bs.abr.2015.10.007

[B40] Hachiya T. Sakakibara H. (2017). Interactions between nitrate and ammonium in their uptake, allocation, assimilation, and signaling in plants. J. Exp. Bot. 68, 2501–2512. 10.1093/jxb/erw449 28007951

[B41] Hakata M. Takahashi M. Zumft W. Sakamoto A. Morikawa H. (2003). Conversion of the nitrate nitrogen and nitrogen dioxide to nitrous oxides in plants. Acta Biotechnol. 23, 249–257. 10.1002/abio.200390032

[B42] Horchani F. Aschi-Smiti S. Brouquisse R. (2010). Involvement of nitrate reduction in the tolerance of tomato (*Solanum lycopersicum* L.) plants to prolonged root hypoxia. Acta Physiol. Plant 32, 1113–1123. 10.1007/s11738-010-0503-0

[B43] Horchani F. Prévot M. Boscari A. Evangelisti E. Meilhoc E. Bruand C. (2011). Both plant and bacterial nitrate reductases contribute to nitric oxide production in *Medicago truncatula* nitrogen-fixing nodules. Plant Physiol. 155, 1023–1036. 10.1104/pp.110.166140 21139086 PMC3032450

[B44] Horn M. A. Schramm A. Drake H. L. (2003). The earthworm gut: an ideal habitat for ingested N_2_O-producing microorganisms. Appl. Environ. Microb. 69, 1662–1669. 10.1128/AEM.69.3.1662-1669.2003 PMC150078 12620857

[B45] Hu H. W. Chen D. He J. Z. (2015). Microbial regulation of terrestrial nitrous oxide formation: understanding the biological pathways for prediction of emission rates. FEMS Microbiol. Rev. 39, 729–749. 10.1093/femsre/fuv021 25934121

[B46] Igamberdiev A. U. Hill R. D. (2009). Plant mitochondrial function during anaerobiosis. Ann. Bot. 103, 259–268. 10.1093/aob/mcn100 18586697 PMC2707300

[B47] Igamberdiev A. U. Bykova N. V. Shah J. K. Hill R. D. (2010). Anoxic nitric oxide cycling in plants: participating reactions and possible mechanisms. Physiol. Plantarum 138, 393–404. 10.1111/j.1399-3054.2009.01314.x 19929898

[B48] Igamberdiev A. U. Ratcliffe R. G. Gupta K. J. (2014). Plant mitochondria: source and target for nitric oxide. Mitochondrion 19, 329–333. 10.1016/j.mito.2014.02.003 24561220

[B49] Jakobs H. H. Froriep D. Havemeyer A. Mendel R. R. Bittner F. Clement B. (2014). The mitochondrial amidoxime reducing component (mARC): involvement in metabolic reduction of N-oxides, oximes and N-hydroxyamidinohydrazones. ChemMedChem 9, 2381–2387. 10.1002/cmdc.201402127 25045021

[B50] Jansson E.Å. Huang L. Malkey R. Govoni M. Nihlén C. Olsson A. (2008). A mammalian functional nitrate reductase that regulates nitrite and nitric oxide homeostasis. Nat. Chem. Biol. 4, 411. 10.1038/nchembio.92 18516050

[B51] John P. Whatley F. R. (1975). *Paracoccus denitrificans* and the evolutionary origin of the mitochondrion. Nature 254, 495. 10.1038/254495a0 235742

[B52] Johnson K. S. Barbehenn R. V. (2000). Oxygen levels in the gut lumens of herbivorous insects. J. Insect Physiol. 46, 897–903. 10.1016/S0022-1910(99)00196-1 10802101

[B53] Kadenbach B. Stroh A. Hüther F. J. Reimann A. Steverding D. (1991). Evolutionary aspects of cytochromec oxidase. J. Bioenerg. Biomembr. 23 (2), 321–334. 10.1007/BF00762225 1646800

[B54] Klepper L. A. (1987). Nitric oxide emissions from soybean leaves during in vivo nitrate reductase assays. Plant Physiol. 85, 96–99. 10.1104/pp.85.1.96 16665692 PMC1054210

[B55] Kobayashi M. Matsuo Y. Takimoto A. Suzuki S. Maruo F. Shoun H. (1996). Denitrification, a novel type of respiratory metabolism in fungal mitochondrion. J. Biol. Chem. 271, 16263–16267. 10.1074/jbc.271.27.1626 8663075

[B56] Koivisto A. Matthias A. Bronnikov G. Nedergaard J. (1997). Kinetics of the inhibition of mitochondrial respiration by NO. FEBS Lett. 417, 75–80. 10.1016/S0014-5793(97)01258-1 9395078

[B57] Kozlov A. V. Staniek K. Nohl H. (1999). Nitrite reductase activity is a novel function of mammalian mitochondria. FEBS Lett. 454, 127–130. 10.1016/S0014-5793(99)00788-7 10413109

[B58] Kroneck P. M. (2018). Walking the seven lines: binuclear copper A in cytochrome c oxidase and nitrous oxide reductase. J. Biol. Inorg. Chem. 23, 27–39. 10.1007/s00775-017-1510-z 29218634

[B59] Lenhart K. Weber B. Elbert W. Steinkamp J. Clough T. Crutzen P. (2015). Nitrous oxide and methane emissions from cryptogamic covers. Global Change Biol. 21, 3889–3900. 10.1111/gcb.12995 26152454

[B60] Lenhart K. Behrendt T. Greiner S. Steinkamp J. Well R. Giesemann A. (2019). Nitrous oxide effluxes from plants as a potentially important source to the atmosphere. New Phytol. 221, 1398–1408. 10.1111/nph.15455 30303249

[B61] Ludwig B. (1987). Cytochrome c oxidase in prokaryotes. FEMS Microbiol. Rev. 3 (1), 41–56. 10.1111/j.1574-6968.1987.tb02451.x

[B62] Lundberg J. O. Weitzberg E. Gladwin M. T. (2008). The nitrate–nitrite–nitric oxide pathway in physiology and therapeutics. Nat. Rev. Drug Discovery 7, 156. 10.1038/nrd2466 18167491

[B63] Maathuis F. J. (2009). Physiological functions of mineral macronutrients. Curr. Opin. Plant Biol. 12, 250–258. 10.1016/j.pbi.2009.04.003 19473870

[B64] Machacova K. Papen H. Kreuzwieser J. Rennenberg H. (2013). Inundation strongly stimulates nitrous oxide emissions from stems of the upland tree Fagus sylvatica and the riparian tree *Alnus glutinosa*. Plant Soil 364, 287–301. 10.1007/s11104-012-1359-4

[B65] Machacova K. Maier M. Svobodova K. Lang F. Urban O. (2017). Cryptogamic stem covers may contribute to nitrous oxide consumption by mature beech trees. Sci. Rep. 7, 13243. 10.1038/s41598-017-13781-7 29038453 PMC5643534

[B66] Maia L. B. Moura J. J. (2015). Nitrite reduction by molybdoenzymes: a new class of nitric oxide-forming nitrite reductases. J. Biol. Inorg. Chem. 20, 403–433. 10.1007/s00775-014-1234-2 25589250

[B67] Miller A. J. Cramer M. D. (2005). “Root nitrogen acquisition and assimilation,” in Root Physiology: from Gene to Function. (Dordrecht: Springer), 1–36.

[B68] Morard P. Silvestre J. Lacoste L. Caumes E. Lamaze T. (2004). Nitrate uptake and nitrite release by tomato roots in response to anoxia. J. Plant Physiol. 161, 855–865. 10.1016/j.jplph.2003.11.003 15310075

[B69] Moreau M. Lee G. II Wang Y. Crane B. R. Klessig D. F. (2008). AtNOS/AtNOA1 is a functional Arabidopsis thaliana cGTPase and not a nitric-oxide synthase. J. Biol. Chem. 283, 32957–32967. 10.1074/jbc.M804838200 18801746 PMC2583314

[B70] Oliveira H. C. Sodek L. (2013). Effect of oxygen deficiency on nitrogen assimilation and amino acid metabolism of soybean root segments. Amino Acids 44, 743–755. 10.1007/s00726-012-1399-3 22990842

[B71] Oliveira H. C. Salgado I. Sodek L. (2012). Involvement of nitrite in the nitrate-mediated modulation of fermentative metabolism and nitric oxide production of soybean roots during hypoxia. Planta 237, 255–264. 10.1007/s00425-012-1773-0 23011570

[B72] Oliveira H. C. Salgado I. Sodek L. (2013). Nitrite decreases ethanol production by intact soybean roots submitted to oxygen deficiency: a role for mitochondrial nitric oxide synthesis? Plant Signal. Behav. 8, e23578. 10.4161/psb.23578 23333978 PMC9583730

[B73] O’Brien J. A. Vega A. Bouguyon E. Krouk G. Gojon A. Coruzzi G. (2016). Nitrate transport, sensing, and responses in plants. Mol. Plant 9, 837–856. 10.1016/j.molp.2016.05.004 27212387

[B74] Pearce L. L. Kanai A. J. Birder L. A. Pitt B. R. Peterson J. (2002). The catabolic fate of nitric oxide the nitric oxide oxidase and peroxynitrite reductase activities of cytochrome oxidase. J. Biol. Chem. 277, 13556–13562. 10.1074/jbc.M109838200 11825895

[B75] Planchet E. Gupta K. J. Sonoda M. Kaiser W. M. (2005). Nitric oxide emission from tobacco leaves and cell suspensions: rate limiting factors and evidence for the involvement of mitochondrial electron transport. Plant J. 41, 732–743. 10.1111/j.1365-313X.2005.02335.x 15703060

[B76] Plouviez M. Wheeler D. Shilton A. Packer M. A. McLenachan P. A. Sanz-Luque E. (2017). The biosynthesis of nitrous oxide in the green alga *Chlamydomonas reinhardtii*. Plant J. 91, 45–56. 10.1111/tpj.13544 28333392

[B77] Plouviez M. Shilton A. Packer M. A. Guieysse B. (2019). Nitrous oxide emissions from microalgae: potential pathways and significance. J. Appl. Phycol. 31, 1–8. 10.1007/s10811-018-1531-1

[B78] Poderoso J. J. Helfenberger K. Poderoso C. (2019). The effect of nitric oxide on mitochondrial respiration. Nitric. Oxide 88, 61–72. 10.1016/j.niox.2019.04.005 30999001

[B79] Rockel P. Strube F. Rockel A. Wildt J. Kaiser W. M. (2002). Regulation of nitric oxide (NO) production by plant nitrate reductase in vivo and in vitro. J. Exp. Bot. 53, 103–110. 10.1093/jexbot/53.366.103 11741046

[B80] Rőszer T. (2012). The biology of subcellular nitric oxide. Dordrecht: Springer. 10.1007/978-94-007-2819-6

[B81] Rusch H. Rennenberg H. (1998). Black alder (*Alnus glutinosa* (L.) Gaertn.) trees mediate methane and nitrous oxide emission from the soil to the atmosphere. Plant Soil 201, 1–7. 10.1023/A:1004331521059

[B82] Sanchez-Cruz P. Alegría A. E. (2009). Quinone-enhanced reduction of nitric oxide by xanthine/xanthine oxidase. Chem. Res. Toxicol. 22, 818–823. 10.1021/tx800392j 19301825 PMC2753477

[B83] Saraste M. Castresana J. (1994). Cytochrome oxidase evolved by tinkering with denitrification enzymes. FEBS Lett. 341, 1–4. 10.1016/0014-5793(94)80228-9 8137905

[B84] Saraste M. (1994). Structure and evolution of cytochrome oxidase. Anton. Leeuw. Int. J. 65, 285–287. 10.1007/BF00872214 7832587

[B85] Senbayram M. Well R. Shan J. Bol R. Burkart S. Jones D. L. (2020). Rhizosphere processes in nitrate-rich barley soil tripled both N_2_O and N_2_ losses due to enhanced bacterial and fungal denitrification. Plant Soil 448, 509–522. 10.1007/s11104-020-04457-9

[B86] Smart D. R. Bloom A. J. (2001). Wheat leaves emit nitrous oxide during nitrate assimilation. P. Natl. Acad. Sci. 98, 7875–7878. 10.1073/pnas.131572798 PMC35435 11427711

[B87] Sparacino-Watkins C. E. Tejero J. Sun B. Gauthier M. C. Thomas J. Ragireddy V. (2014). Nitrite reductase and nitric-oxide synthase activity of the mitochondrial molybdopterin enzymes mARC1 and mARC2. J. Biol. Chem. 289, 10345–10358. 10.1074/jbc.M114.555177 24500710 PMC4036158

[B88] Stanton C. L. Reinhard C. T. Kasting J. F. Ostrom N. E. Haslun J. A. Lyons T. W. (2018). Nitrous oxide from chemodenitrification: A possible missing link in the Proterozoic greenhouse and the evolution of aerobic respiration. Geobiology 16, 597–609. 10.1111/gbi.12311 30133143

[B89] Stief P. Poulsen M. Nielsen L. P. Brix H. Schramm A. (2009). Nitrous oxide emission by aquatic macrofauna. P. Natl. Acad. Sci. 106, 4296–4300. 10.1073/pnas.0808228106 PMC2651200 19255427

[B90] Stoimenova M. Igamberdiev A. U. Gupta K. J. Hill R. D. (2007). Nitrite-driven anaerobic ATP synthesis in barley and rice root mitochondria. Planta 226, 465–474. 10.1007/s00425-007-0496-0 17333252

[B91] Sugiura M. Georgescu M. N. Takahashi M. (2007). A nitrite transporter associated with nitrite uptake by higher plant chloroplasts. Plant Cell Physiol. 48, 1022–1035. 10.1093/pcp/pcm073 17566055

[B92] Syakila A. Kroeze C. (2011). The global nitrous oxide budget revisited. GGMM 1, 17–26. 10.3763/ghgmm.2010.0007

[B93] Taylor C. T. Moncada S. (2010). Nitric oxide, cytochrome C oxidase, and the cellular response to hypoxia. Arterioscl. Throm. Vas. 30, 643–647. 10.1161/ATVBAHA.108.181628 19713530

[B94] Thomson A. J. Giannopoulos G. Pretty J. Baggs E. M. Richardson D. J. (2012). Biological sources and sinks of nitrous oxide and strategies to mitigate emissions. Philos. T. R. Soc B. 367, 1157–1168. 10.1098/rstb.2011.0415 PMC3306631 22451101

[B95] Tian H. Yang J. Lu C. Xu R. Canadell J. G. Jackson R. B. (2018). The global N_2_O model intercomparison project. B. Am. Meteorol. Soc. 99, 1231–1251. 10.1175/BAMS-D-17-0212.1

[B96] Tischner R. Planchet E. Kaiser W. M. (2004). Mitochondrial electron transport as a source for nitric oxide in the unicellular green alga *Chlorella sorokiniana*. FEBS Lett. 576, 151–155. 10.1016/j.febslet.2004.09.004 15474028

[B97] van der Leij M. Smith S. Miller A. (1998). Remobilisation of vacuolar stored nitrate in barley root cells. Planta 205, 64–72. 10.1007/s004250050297

[B98] Vanecko S. Varner J. E. (1955). Studies on nitrite metabolism in higher plants. Plant Physiol. 30:388. 10.1104/pp.30.4.388 16654795 PMC540672

[B99] Vartapetian B. B. Andreeva I. N. Generozova I. P. Polyakova L. II Maslova I. P. Dolgikh Y. II (2003). Functional electron microscopy in studies of plant response and adaptation to anaerobic stress. Ann. Bot. 91, 155–172. 10.1093/aob/mcf244 12509337 PMC4244998

[B100] Voesenek L. A. Sasidharan R. Visser E. J. Bailey-Serres J. (2016). Flooding stress signaling through perturbations in oxygen, ethylene, nitric oxide and light. New Phytol. 209, 39–43. 10.1111/nph.13775 26625347

[B101] von Wirén N. Gazzarrini S. Gojon A. Frommer W. B. (2000). The molecular physiology of ammonium uptake and retrieval. Curr. Opin. Plant Biol. 3, 254–261. 10.1016/S1369-5266(00)80074-6 10837267

[B102] Wany A. Kumari A. Gupta K. J. (2017). Nitric oxide is essential for the development of aerenchyma in wheat roots under hypoxic stress. Plant Cell Environ. 40, 3002–3017. 10.1111/pce.13061 28857271

[B103] Wany A. Gupta A. K. Kumari A. Mishra S. Singh N. Pandey S. (2019). Nitrate nutrition influences multiple factors in order to increase energy efficiency under hypoxia in Arabidopsis. Ann. Bot. 123, 691–705. 10.1093/aob/mcy202 30535180 PMC6417481

[B104] Ward N. D. Indivero J. Gunn C. Wang W. Bailey V. McDowell N. G. (2019). Longitudinal gradients in tree stem greenhouse gas concentrations across six Pacific Northwest coastal forests. J. Geophys. Res.- Biogeo. 124, 1401–1412. 10.1029/2019JG005064

[B105] Weathers P. J. Niedzielski J. J. (1986). Nitrous oxide production by cyanobacteria. Arch. Microbiol. 146, 204–206. 10.1007/BF00402352

[B106] Weathers P. J. (1984). N_2_O evolution by green algae. Appl. Environ. Microb. 48, 1251–1253. 10.1128/AEM.48.6.1251-1253.1984 PMC241719 16346687

[B107] Welch B. Gauci V. Sayer E. J. (2019). Tree stem bases are sources of CH_4_ and N_2_O in a tropical forest on upland soil during the dry to wet season transition. Global Change boil. 25, 361–372. 10.1111/gcb.14498 30367532

[B108] Wen Y. Corre M. D. Rachow C. Chen L. Veldkamp E. (2017). Nitrous oxide emissions from stems of alder, beech and spruce in a temperate forest. Plant Soil 420, 423–434. 10.1007/s11104-017-3416-5

[B109] Wendehenne D. Pugin A. Klessig D. F. Durner J. (2001). Nitric oxide: comparative synthesis and signaling in animal and plant cells. Trends Plant Sci. 6, 177–183. 10.1016/S1360-1385(01)01893-3 11286923

[B110] Woehle C. Roy A. S. Glock N. Wein T. Weissenbach J. Rosenstiel P. (2018). A novel eukaryotic denitrification pathway in foraminifera. Curr. Biol. 28, 2536–2543. 10.1016/j.cub.2018.06.027 30078568 PMC6783311

[B111] Wulff A. Oliveira H. C. Saviani E. E. Salgado I. (2009). Nitrite reduction and superoxide-dependent nitric oxide degradation by Arabidopsis mitochondria: influence of external NAD (P) H dehydrogenases and alternative oxidase in the control of nitric oxide levels. Nitric. Oxide 21, 132–139. 10.1016/j.niox.2009.06.003 19576290

[B112] Zhao X. J. Sampath V. Caughey W. S. (1995). Cytochrome c oxidase catalysis of the reduction of nitric oxide to nitrous oxide. Biochem. Bioph. Res. Co. 212, 1054–1060. 10.1006/bbrc.1995.2076 7626092

[B113] Zhao L. Liu F. Crawford N. M. Wang Y. (2018). Molecular regulation of nitrate responses in plants. Int. J. Mol. Sci. 19, 2039. 10.3390/ijms19072039 PMC6073361 30011829

